# SARS-CoV-2 Infection Triggers Auto-Immune Response in ARDS

**DOI:** 10.3389/fimmu.2022.732197

**Published:** 2022-01-28

**Authors:** Pablo Juanes-Velasco, Alicia Landeira-Viñuela, Marina L. García-Vaquero, Quentin Lecrevisse, Raquel Herrero, Antonio Ferruelo, Rafael Góngora, Fernando Corrales, Javier De Las Rivas, Jose A. Lorente, Ángela-Patricia Hernández, Manuel Fuentes

**Affiliations:** ^1^ Department of Medicine and Cytometry General Service-Nucleus, CIBERONC, Cancer Research Centre (IBMCC/CSIC/USAL/IBSAL), Salamanca, Spain; ^2^ Department of Critical Care Medicine, Hospital Universitario de Getafe, Madrid, Spain; ^3^ CIBER de Enfermedades Respiratorias, Instituto de Investigación Carlos III, Madrid, Spain; ^4^ Fundación de Investigación Biomédica del Hospital Universitario de Getafe, Madrid, Spain; ^5^ Functional Proteomics Laboratory, National Center for Biotechnology, Consejo Superior de Investigaciones Científicas, Madrid, Spain; ^6^ PROTEORED-ISCIII, Red Nacional de Laboratorios de Proteomica-ISCIII, Madrid, Spain; ^7^ Bioinformatics and Functional Genomics Group, Cancer Research Center (IBMCC, CSIC/USAL/IBSAL), Consejo Superior de Investigaciones Científicas & University of Salamanca, Salamanca, Spain; ^8^ Universidad Europea de Madrid, Madrid, Spain; ^9^ Proteomics Unit, Cancer Research Centre (IBMCC/CSIC/USAL/IBSAL), Salamanca, Spain

**Keywords:** SARS-CoV-2, COVID-19, ARDS, auto-antibodies, antigen, acute-phase proteins, proteomics, microarrays

## Abstract

Acute respiratory distress syndrome (ARDS) is a severe pulmonary disease, which is one of the major complications in COVID-19 patients. Dysregulation of the immune system and imbalances in cytokine release and immune cell activation are involved in SARS-CoV-2 infection. Here, the inflammatory, antigen, and auto-immune profile of patients presenting COVID-19-associated severe ARDS has been analyzed using functional proteomics approaches. Both, innate and humoral responses have been characterized through acute-phase protein network and auto-antibody signature. Severity and sepsis by SARS-CoV-2 emerged to be correlated with auto-immune profiles of patients and define their clinical progression, which could provide novel perspectives in therapeutics development and biomarkers of COVID-19 patients. Humoral response in COVID-19 patients’ profile separates with significant differences patients with or without ARDS. Furthermore, we found that this profile can be correlated with COVID-19 severity and results more common in elderly patients.

## 1 Introduction

Coronaviruses (CoV) are a family of single-stranded positive RNA viruses with high diversity. This family is classified into three groups: *Alphacoronavirus*, *Betacoronavirus* and *Gammacoronavirus.* The first two infect mammals and the third are avian viruses. Currently, seven types of CoVs are known to be capable of infecting humans, two *Alphacoronavirus* (CoV-229E and CoV-NL63) and five *Betacoronavirus* (CoV-HKU1, CoV-OC43, SARS-CoV, MERS-CoV, and SARS-CoV-2) (1–3).

It is interesting to note that all CoVs have common characteristics. Their structural proteins include the spike glycoprotein (S), responsible for virus–host cell interaction; envelope (E) and membrane (M), accountable for the formation of the viral envelope and nucleocapsid (N), in charge of forming a helical complex that interacts with the M protein during the virion assembly process. Also, there are non-structural proteins (involved in virus transcription and replication) and other accessory proteins. During the CoV infection, the S protein is cleaved into two subunits (S1 and S2). S1 subunit contains the receptor binding domain (RBD) (responsible for the trimer organization of the S protein), whereas the S2 subunit has the fusion machinery (2–4).

While CoV-229E, CoV-NL63, CoV-OC43, and CoV-HKU1 exhibit low pathogenicity; SARS-CoV, MERS-CoV, and SARS-CoV-2 cause atypical pneumonias that can lead to acute respiratory distress syndrome (ARDS). As is well known, SARS-CoV 2 is currently considered a global health emergency. This CoV was discovered in December 2019 (COVID-19) due to an emerging outbreak in Wuhan (China). The main clinical features of patients of COVID-19 disease are very heterogeneous and range from asymptomatic disease to severe atypical pneumonia (1, 3). The underlying factors for this heterogeneous presentation have been an area of intense research. Understanding these factors would help in stratifying patients that are at high risk of adverse outcomes and also shedding light in the pathophysiology of this disease to meet the urgent need for a targeted and rational treatment design against this serious global affliction.

As in any pathogen infection, the immune system plays a crucial role in the development and consequences of COVID-19. Both the innate and adaptive responses triggered by SARS-CoV 2 have attracted the attention of the scientific community (5). In general terms, the inflammatory response is the hallmark of the innate immune system. This non-specific response to environmental assaults such as pathogens can also be induced by persistent tissue damage and the release of damage-associated patterns (DAMPs). The resultant alterations in homeostasis manifest themselves as the imbalances in crosstalk plasmatic cascades such as coagulation, fibrinolysis, or the complement system (6).

With regards to the adaptive response, serum antibodies are essential components of specific immunity but are also involved in the pathogenesis of many autoimmune diseases, allergies, and oncopathologies. While a lot is known about the mechanism of host antibody production following pathogen or infectious exposure or vaccination, the induction of autoantibodies (AABs) in many other diseases still remains to be clearly elucidated (7, 8).

Knowledge of autoantibodies dates back to the 1940s. This type of antibody is produced in the absence of foreign antigen in reaction to the host’s own components. These autoantibodies are characterized as IgM isotype antibodies, possessing polyreactivity and high antigen avidity. In genetically susceptible individuals, infection and other environmental factors have been described as factors which trigger immune responses by different mechanisms, namely, cytokine production and release, stimulation of toll-like receptors and other pattern recognition receptors, the release of self-antigens by damaged cells and tissues, and/or molecular mimicry (9, 10). On the other hand, the production of molecular patterns associated with damage and pathogen-associated molecular patterns after the entry of microorganisms is also one of the causes of the generation of autoantibodies, which seems to be highly correlated with this type of response in SARS-CoV-2 infection (11).

However to date, it is still unknown about the spectrum of AAB responses and kinetics of AAB induction during acute infection and systemic inflammation (12). Regarding COVID-19, ARDS and sepsis are one of the most serious clinical complications. ARDS and sepsis are acute inflammatory conditions associated with high morbidity and mortality, often involving multiple organ failure. ARDS is caused by a wide variety of infectious or inflammatory stimuli to the lung that may occur by direct (i.e., pneumonia) or indirect injury (i.e., peritonitis). The pathological hallmarks of ARDS are diffuse alveolar damage manifested by disruption of alveolar capillary interface, and also the accumulation of immune cells (innate and adaptive immune cells) and protein-rich exudates in the alveolar spaces (13). COVID-19 patients have elevated levels of cytokines and several inflammatory mediators (such as IL-6, TNF-α or IL-8, among others) in lung proximal fluids and peripheral blood. SARS-CoV-2 infections cause local and systemic inflammatory responses; however, in sepsis, there is a rapid shift towards an anti-inflammatory immunosuppressive state, loss of dendritic cells, and low B cells and CD4^+^ lymphocyte counts; on the contrary, the cytokine storm with systemic consequences is noteworthy (14). In addition, the presence of AABs has been reported in COVID-19 patients and its correlation with disease outcome (15).

In this study, a systematic evaluation of humoral responses has been carried out by functional proteomics, in order to describe a mechanism and time course for the rapid induction of AABs seen in ARDS and sepsis in SARS-CoV-2 patients, which could provide novel insights in treatment, diagnosis, and prognosis. Herein, a multiplex array for simultaneous detection of acute phase components and AABs (against a panel of potential autoantigens) have been designed and validated.

## 2 Materials and Methods

### 2.1 Materials

All reagents were of analytical grade and were used as received without further purification. Sodium acetate (AcONa), isopropyl alcohol, ethanol 96%, 3-(2-Aminoethylamino)-propyldimethoxymethylsilane (MANAE) (≥95.0%), BS3 (bis(sulfosuccinimidyl)suberate), dimethyl sulfoxide (DMSO), glycerol 85%, bovine serum albumin (BSA), Tween™ 20, Hybriwell sealing system, Lysogeny broth (LB) medium, Grace Bio-Labs ProPlate^®^ microarray system, Grace Bio-Labs ProPlate^®^ clips for microarray systems (Sigma-Aldrich, St. Louis/MO, USA); SuperBlock™ Blocking Buffer (TBS), Pierce™ BCA Protein Assay Kit, EZ-Link NHS-PEG4 Biotin., Blocker™ BSA (10%) in PBS, Quant-it Pico Green dsDNA Assay Kit, Microscope slides (76 × 26 mm), Mseries Lifterlip y Lifterslip™ coverslips, Coronavirus Ig Total Human 11-Plex ProcartaPlex™ Panel (Thermo Scientific, Rockford/IL, USA); Microarray-Specific 384-well Microplates, JetStar™, Optimum Microarray Printing Buffer C (ArrayJet, Roslin, UK); Corning^®^ 96-well Black Flat Bottom Polystyrene Not Treated Microplate (Corning, Somerville, Massachusetts, USA); Cytiva Amersham™ Streptavidin-Fluor Cy3 (GE-Healthcare, Little Chalfont, Buckinghamshire, UK); MAGPIX^®^ Drive Fluid, 4 pack (Merck KGaA, Darmstadt, Germany); TnT^®^ Coupled Reticulocyte Lysate System kit, Pure Yield plasmid miniprep system (Promega, Madison/WI, USA); TSA Individual Cyanine 3 Tyramid Reagent Pack (PerkinElmer, Waltham/MA, USA). Antibodies used in this report are detailed in **Table S1**.

### 2.2 Equipment and Software

ArrayJet^®^ Printer Marathon v1.4, JetSpyder™ 12 samples, JetStar™ (ArrayJet, Roslin, UK); Scanner SensoSpot Fluorescence (Miltenyi Imaging GmbH, Radolfzell, Germany); Orbital shaker (FALC Instruments S.r.l.; Treviglio, Italy); Fisherbrand™ Microplate Vortex Mixers (Fisherbrand™, EEUU); T100 Thermal Cycler (Biorad, Hercules/CA, USA); Magnetic 96-Well Separator, Digital Dry Block Heater (Thermo Scientific, Rockford/IL, USA); GenePix^®^ Pro Microarray Analysis Software (Molecular Devices, San Jose/CA, USA); R statistics software (R Foundation for Statistical Computing, Vienna, Austria. http://www.R-project.org/); MAGPIX^®^ System of xMAP^®^ instruments and xPONENT^®^ Software (Luminex Corporation, Austin, Texas, USA).

### 2.3 Patients and Samples

#### 2.3.1 Cohort 1

Plasma samples from 20 patients diagnosed with COVID-19 by RT-PCR and 10 healthy donors (COVID-19 negative) were collected in the University Hospital of Salamanca (HUS, Salamanca, Spain) and deposited in the Spanish National DNA Biobank (Banco Nacional de ADN, University of Salamanca). In all cases, each patient gave informed consent prior to entering the study and was subsequently approved by the HUS ethics committee. The most relevant clinical and laboratory information are summarized in **Tables S1, S2**.

#### 2.3.2 Cohort 2

Plasma samples from 76 patients diagnosed with COVID-19 by RT-PCR were collected in the University Hospital of Miguel Servet (HUMS, Zaragoza, Spain). The most relevant clinical information of the patients is summarized in **Tables S3, S4**.

### 2.4 Antigen Profiling of Different Coronavirus Strains

Kit ProcartaPlex Human Coronavirus Ig Total Panel 11-plex was used for antigen study as previously reported using xMAP’s methodology (16). All plasma samples were incubated in Corning^®^ 96 Well Solid Polystyrene Microplate together with the standard samples for relative quantification, medium and low control CoV and Assay Buffer as blank. Standard serial dilution was made in PCR 8-Tube Strip. All soluble immunoglobulins (S1 protein for all CoV and for SARS-CoV-2 in addition to the spike trimer -S1 + S2-, RBD and N proteins) are captured with a Bead mix. All washes were carried out with Wash Buffer diluted in deionized water and used a Magnetic 96-Well Separator. Detection antibody was made with Ig total Det Antibody diluted with Detection Ab Diluent (1×). Acquisition was made in Reading Buffer with xMAP^®^ instruments and the MAGPIX^®^ System software was used for analysis. All incubation steps were made in agitation with Fisherbrand™ Microplate Vortex Mixers and cover the plate with Plate Cover and Black Microplate Lid.

### 2.5 Detection of Acute-Phase Proteins (APPs) by Affinity Proteomics

#### 2.5.1 APPs Array Design

Based on previous reports (17), protein array content was designed with 21 different antibodies (**Table S5**) targeting 21 different APPs. Each antibody was resuspended in PBS employing low concentrations from stock solution (ten-fold difference between them). Subsequently, antibody aliquots were diluted 1:1 (v/v) in Arrayjet Printing buffer C, according to ArrayJet Printer Marathon v1.4 specifications. Slide-out has 7 identical subarrays with 432 spots, each antibody is printed in triplicate. Positive (Cy3, anti-biotin antibody and biotin) and negative (GST-antibody, PBS, clean buffer, and printing buffer) as controls were also included in each subarray. A total of 6 serum samples were analyzed per array. Antibodies were deposited on a chemically activated surface prepared according to previous reports (18) with ArrayJet Printer Marathon v1.4. Eventually, printed arrays were packed and stored protected from light in dry atmosphere at room temperature (RT) until assayed.

##### 2.5.1.1 Sera Biotinylation

Following the protocol described previously by Henjes et al. (17, 19), plasma proteins (100 µg) were biotinylated by incubation with 0.78 mg/ml of NHS-PEG4-biotin (12 µl in DMSO) for 2 h at 4°C. Biotinylation reactions were stopped with 0.5 M Tris–HCl (pH 8) obtaining a final concentration of biotin 1:200 (v/v) in each sample.

##### 2.5.1.2 APPs Screening

Firstly, 100 μl of biotinylated serum 1:100 (v/v) in SuperBlock^®^ BSA were prepared. Epitope retrieval was performed according to a previously described method (20). Microarrays were blocked and washed with distilled water (3 times, 5 min). Then, samples were incubated overnight at 4°C with slight shaking. After that, the arrays were washed with PBS with Tween (PBST) (0.05%) (3×, 5 min) and revealed using 1:200 (v/v) Cy3-Streptavidin for 30 min. Finally, arrays were washed with PBS (3×, 5 min) and distilled water, dried and scanned.

##### 2.5.1.3 Image Acquisition

Array images were obtained by Scanner SensoSpot Fluorescence. The TIFF images generated by array scanning were analyzed using GenePix Pro 6.0. software. Parameters were set to quantify light intensity values at Cy3 (λ = 532 nm) (21).

### 2.6 AAB Profiling by Nucleic Acid-Programmable Protein Array (NAPPA)

#### 2.6.1 NAPPA Array for AAB Profiling

Based on previous reports (22), NAPPA was built with 30 cDNAs encoding ARDS AABs in triplicate and positive (Cy3, MasterMix) and negative (GST-antibody, PBS, bovine serum albumin (BSA), Bis-(sulfosuccinimidyl) suberate (BS3), clean buffer, and printing buffer) controls (**Table S6**). The design and distribution of the cDNAs on the arrays was as follows: 6 subarrays, each subarray with 144 spots, deposited on a chemically activated surface prepared accordingly with ArrayJet Printer Marathon v1.4.

All the cDNAs (pANT7-cGST plasmids) from DNASU (https://dnasu.org/DNASU/Home.do) were sequence validated. DNA prep for NAPPA arrays were carried out as previously described by Manzano et al. (23). Purified cDNAs were precipitated by the addition of 0.8× volumes of isopropanol and centrifugation at 4,000*g* for 30 min at RT. Precipitated cDNAs were then washed with 80% (v/v) ethanol and allowed to air-dry. cDNA (15 µg) of each precipitated plasmid were dissolved in 15 µl of MasterMix solution containing 33.3 mg/ml BSA, 2.5 mg/ml rabbit polyclonal anti-GST antibody and 2 mM BS3 and transferred to a 384-plate with 15 µl of glycerol 47% (v/v).

#### 2.6.2 NAPPA Performance

##### 2.6.2.1 Quality Control (QC)

In all the NAPPA assays, to check the deposition of the cDNA on the microarray surface, microarrays were blocked, and cDNA staining was carried out by incubating each microarray with 200 μl of picogreen solution diluted by 1:600 (v/v). Additionally, to observe *in situ* protein expression, arrays were blocked and incubated with IVTT system kit (TnT^®^ Coupled Reticulocyte Lysate System kit, Promega) and analyzed as previously described (23, 24).

##### 2.6.2.2 Serum Screening

For serum antibody screening, microarrays were blocked, washed with distilled water and dried with compressed nitrogen gas. Proteins were then expressed using the protocol for the IVTT system. The master mix for this IVTT system was prepared with 200 μl of reticulocyte lysate containing 16 μl of TNT buffer, 8 μl of T7 polymerase, 4 μl of -Met, 4 μl of -Leu or -Cys and 168 μl of DEPC water, and used following the manufacturer’s instructions. The IVTT system was incubated on the microarrays, using HybriWell gaskets. The microarrays were incubated for 90 min at 30°C and 30 min at 15°C for protein expression and capture by the polyclonal rabbit anti-GST antibody. The HybriWell gaskets were then removed, and the arrays were washed with MixMilk (PBS, 0.5% milk powder and 0.2% Tween20) 1 h on an orbital shaker. The microarrays were washed with distilled water and both cohorts were incubated in each subarray (1:100 (v/v) in MixMilk as previously described (25) at 4°C in rotation overnight. The next day, the microarrays were washed 3× with PBST 0.05%. First, microarrays were then incubated with HRP-linked anti-human IgG for 30 min at a dilution of 1:200 (v/v) and then washed again three times with PBST 0.05%. Secondly, microarrays were incubated with 200 μl/microarray of tyramide signal amplification reagent for 5 min at RT. Microarrays were then washed three times with PBST 0.05%, and then once with distilled water and dried by centrifugation.

##### 2.6.2.3 Image Acquisition

As described above, array images were obtained by Scanner SensoSpot Fluorescence. The TIFF images generated by array scanning were analyzed using GenePix Pro 6.0. software. Parameters were set to quantify light intensity values at Cy3 (λ = 532 nm) (21).

### 2.7 Bioinformatics Analysis

#### 2.7.1 APPs and AAB Microarray Data Pre-Processing and QC

The fluorescence signal retrieved from images processed in the previous section was corrected by subtracting background signal and then transformed into Z score as described in previous reports (26, 27). Overall raw fluorescence and log_2_ (Z score) density distribution were compared to validate signal correction at each microarray employed. Principal Component Analysis (PCA) was performed to discard any microarray-wise batch effect (**Figure S1**). Data processing and analysis were performed in R environment (28).

#### 2.7.2 Biostatistics and Data Visualization

Volcano plots illustrate the statistical significance of Z score ratio changes at any two defined conditions. Z ratio is calculated by subtracting the mean Z score in condition A and mean Z score in condition B and then dividing it by the overall standard deviation of Z score in conditions A and B as previously described (29). Volcano plot Y-axis represents the statistical significance of Z score mean difference in conditions A and B–Wilcoxon Rank Sum test, -log_2_ (p-value)-. Canonical biplot is a visualization technique extensively applied to interpret Principal Component Analysis (PCA). The biplots presented in this work draw both observation and variables—patient samples and microarray proteins respectively—as dots and directed vectors. The vector size and direction indicate the discriminatory power of protein variables at the first two Principal Components. Importantly, the direction of vector variables at biplot can also reveal correlations between sets of protein variables and therefore, corroborate the trends observed in Volcano plots.

All the dendograms depicted in the heat maps presented in this work were generated applying the Euclidean distance and Complete-linkage clustering method. Protein microarray profile of AAB for IL2RB, SFTPD, TNFRSF1B, and ANGPT2 was employed to generate a Random Forest (RF) classification model of ARDS prognosis. RF performance was evaluated by calculating AUC and ROC curves when classifying individuals at cohort 2 with mild symptoms (no ICU) and patients admitted to ICU. Additionally, the complete AAB protein microarray profile was used to generate a series of Random Forest (RF) classification models of ARDS prognosis. RF and ROC curves were generated using random Forest and EPI R packages -ntry = 2, ntree = 500- (30, 31). The plots presented in this work were generated using *ggplot2*, *ggpubr*, *ggbiplot*, *Epi*, *ComplexHeatmap*, and *pathview* R packages (32–36).

Additionally, AAB protein microarray profile was used to generate a series of Random Forest (RF) classification models of ARDS prognosis. RF performance was evaluated calculating AUC and ROC curves when classifying individuals with mild symptoms [no intensive care unit (ICU)] and patients admitted to ICU. RF and ROC curves were generated using RF and EPI R packages -ntry = 2, ntree = 500- (30, 36).

## 3 Results

In the search to cross-check all the immune profiles of patients with ARDS in COVID-19, this study has begun by analyzing the antigenic profile related not only to SARS-CoV-2, but also to other CoVs to collect a complete antigenic profile for this type of viruses. Next, the pattern of acute phase proteins was outlined as indicative of the state of the innate immune response and finally, the profile of AABs related to ARDS was characterized. The use of multiplex assay has allowed the high throughput study of each of the parameters individually and also the global assessment of all in the immune response associated with ARDS associated with COVID-19.

### 3.1 SARS-CoV-2 Antigen Profiling

**Figure 1:**
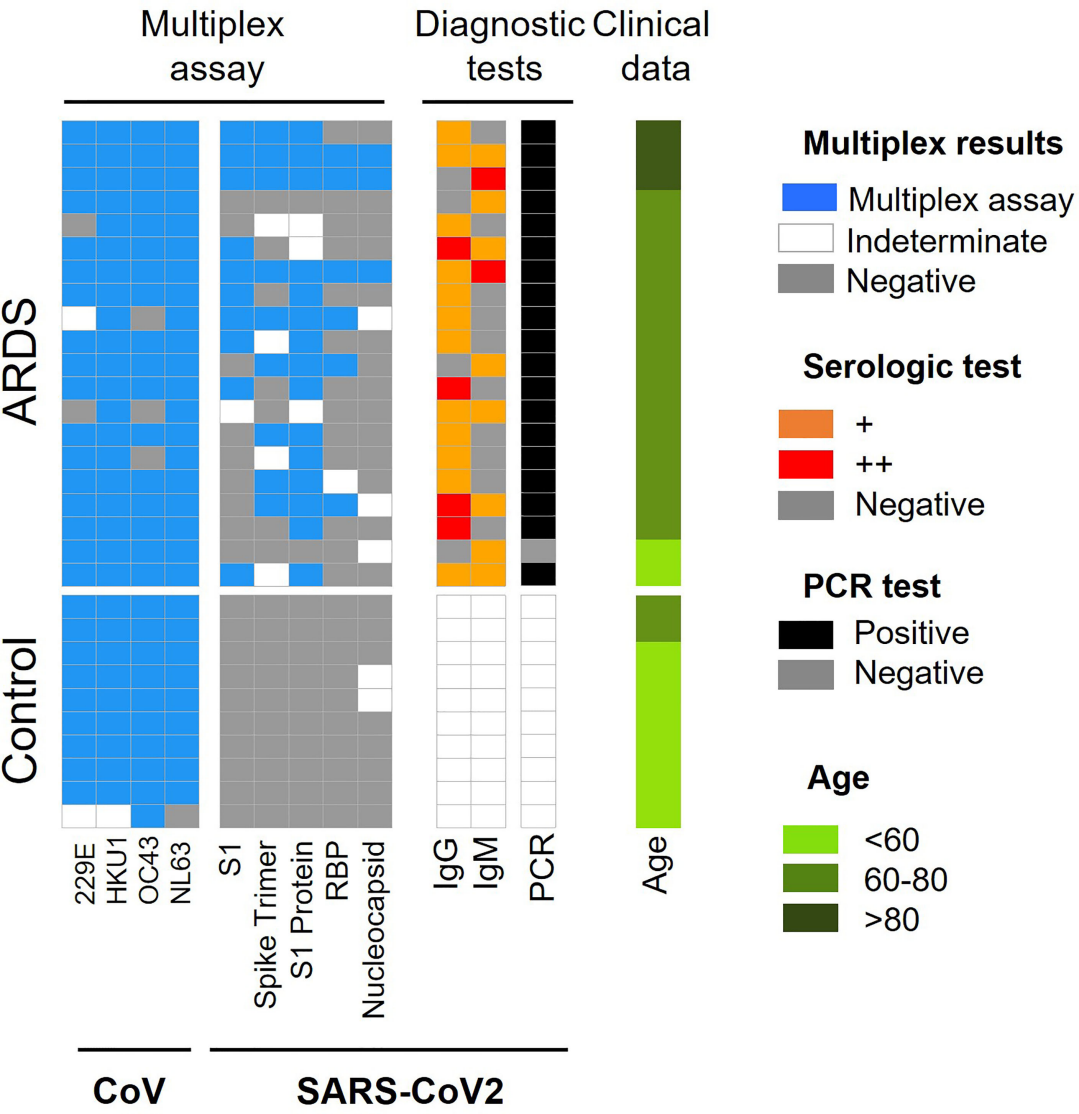
Summary of CoV and SARS-CoV-2 antigen multiplex assay and diagnostic tests employed in ARDS cohort 1. Categorical heat map summarizing the results obtained for SARS-CoV-2 and other coronaviruses (CoV) antigen multiplex assay (blue), PCR (black) and serological diagnostic tests employed for ARDS cohort 1. The serologic test distinguishes high and very high levels of IgG and IgM antibodies (orange and red color, respectively). The samples were separated by clinical symptoms and ordered according to age (green color scale).

Antigenic multiplex assay for other coronaviruses (**Figure 1**) indicates that 87% has S1 protein antibodies for CoV-229E, 97% for CoV-HKU1, 90% for CoV-OC43, 97% for CoV-NL63, and 33% for SARS-CoV.

When analyzing these data according to ± ARDS diagnosis, it is obtained that: i.) ARDS negative: 90% of samples have antibodies for CoV-229E, CoV-HKU1and CoV-NL63 and 100% have antibodies for CoV-OC43, 97% for SARS-CoV. ii.) ARDS positive: 85% of analyzed serum was positive for CoV-229E and CoV-OC43, 100% were positive for V-HKU1 and CoV-NL63 and 50% of them were positive for SARS-CoV proteins (**Figure 1**, **Table 1** and **Table S7**).

**Table 1:** Data summary of cohort 1 for SARS-CoV-2 Antigen Profiling.

Antigenic Multiplex Assay				Serologic test			PCR test
Spike Trimer	S1 Protein	RBD	Nucleocapsid	IgG	IgM	IgG and IgM	
45%	75%	30%	15%	80%	50%	12%	95%

For SARS-CoV-2, it is observed that the protein with the highest antigenic capacity is the S1 protein, present in 75% of the COVID-19 patients. This is followed by the trimeric form of the S1 protein in 45% of samples, RBD in 30% of samples and nucleocapsid in 15% of samples (**Figure 1**). Approximately 80% of COVID-19 patients and with ARDS have IgG antibodies for protein N and 50% presented Ig M. Both immunoglobulins were present in 30% COVID-19 patients (**Figure 1**).

Furthermore, when we study the Wilcoxon mean for ± COVID-19 patients, significant differences between coronavirus existence and ARDS diagnosis are not observed. However, there are significant differences between the antibodies against SARS-CoV-2 proteins (Spike, S1 protein, RBD, and Nucleocapsid) (**Figure S1**).

### 3.2 Evaluation of Humoral Responses in ARDS in COVID-19 Patients

As pointed out above, innate and adaptive humoral immune responses play a key role in the diagnosis and evolution of COVID-19 patients. Once the antigen serologic profile against SARS-CoV-2 and other COVs have been characterized in all the included patients, the analysis of APPs as markers of the innate immune response and the profile of AABs related to ARDS as part of the adaptive immune response has been carried out.

#### 3.2.1 APPs Profiling in COVID-19-Associated ARDS Patients

**Figure 2:**
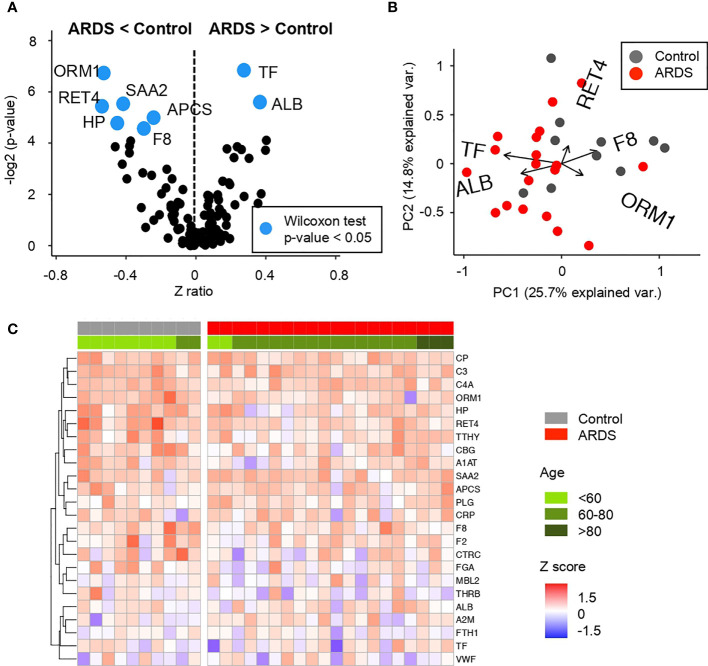
Comparative analysis of the Z scores between control and ARDS patients in the cohort 1 obtained from the microarray for Acute Phase reactants **(A)** Volcano plot summarizing the statistical significance of Z score ratios between control and ARDS patients in the cohort 1 (X-axis). The difference between means was evaluated at 1:500 and 1:5,000 dilutions applying Wilcoxon Rank Sum test (Y-axis). Acute Phase reactants showing statistically significant mean differences are highlighted in blue and larger dots. **(B)** Canonical biplot representing the PCA of the microarray for Acute Phase reactants both at 1:500 and 1:5,000 dilutions employed in the cohort 1. Dots represent samples and vectors the protein variable contribution to the first two principal components at X and Y-axes. The biplot only highlights the most exemplary protein variables. **(C)** Heat map describing Z score values obtained from the microarray for Acute Phase reactants in 1:5,000 dilution in the cohort 1. Samples are separated by clinical symptoms (red and gray labeled columns) and ordered according to patient age (green color scale).

Different inflammatory crosstalking cascades are generically referred to as APPs. Processes that lead up to sepsis seem to be a result of acute activation of these cascades as an alarm signal for the immune system. It might seem that the decrease of the antigenic stimulus or the tissue damage repair could lead to a rapid normalization of APP levels. However, it has been shown that APPs can remain chronically activated after prolonged sepsis. Even sepsis processes can lead to periods of immunosuppression after persistent inflammation (37). In this work, we designed a multiplex platform for the detection of APPs. The relationship between the cascades and the specific APPs is known so the aim of this part of the work is taking the set of APPs, to find biomarkers that can help in the detection and prognosis for COVID-19. Very interesting results about the network established by the APPs have been detected thanks to the new high-throughput screening platform. Significantly activated APPs have been observed in patients with COVID-19 and ARDS disease (**Figure 2** and **Figures S3, S4**).

**Figure 3:**
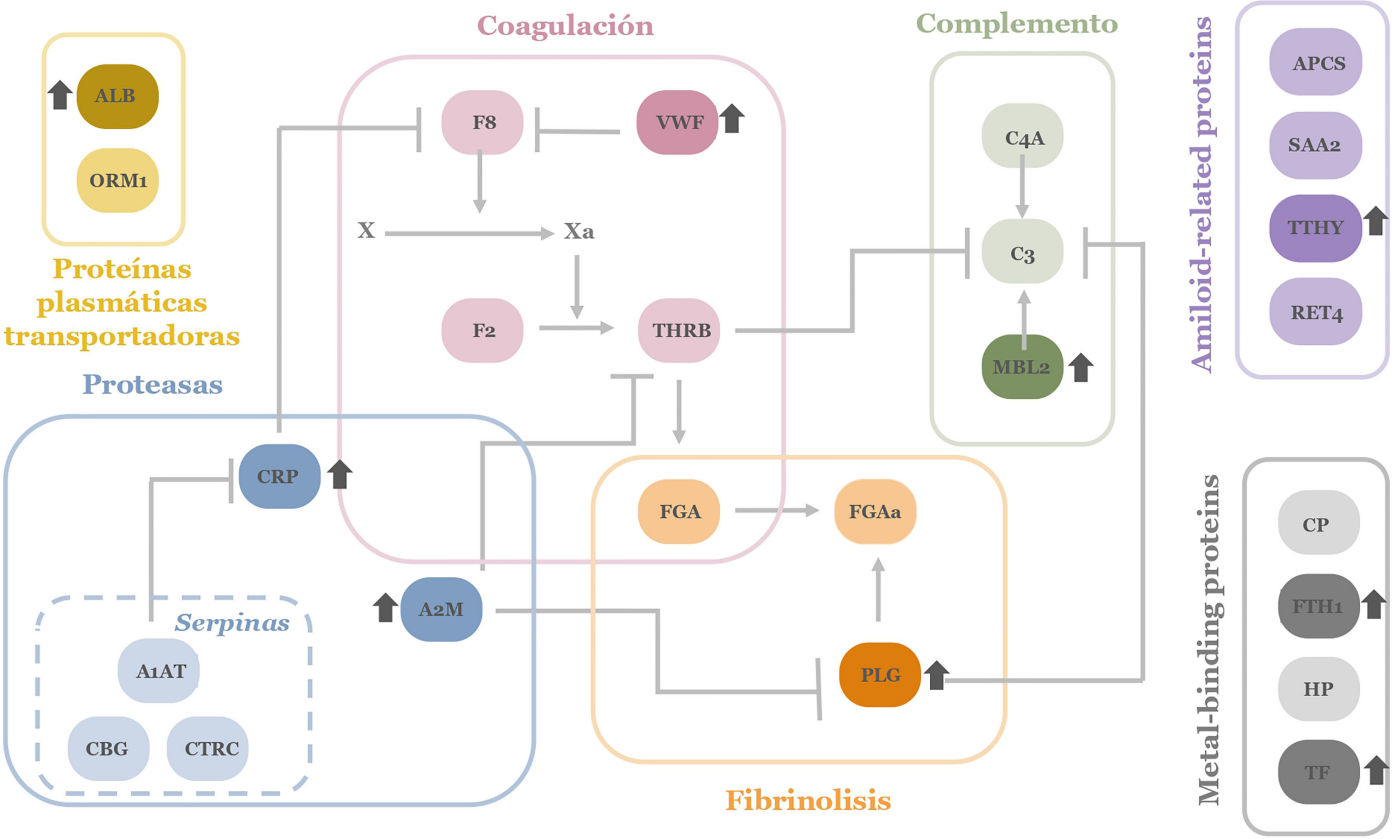
APPs network investigated in microarray screening. The colors correspond to the different physiological processes. In darker colors those proteins that appear more elevated in patients with ARDS in the pathology of COVID-19 are depicted. ALB, Albumin; SAA, serum amyloid A; APCS, serum amyloid P;THBR, thrombin; CP, ceruloplasmin; VWF, von Willebrand factor; C3, C3 complement factor; C4A, C4 complement factor; F8, factor VIII; FTH1, ferritin; FGA, fibrinogen; HP, haptoglobin; MBL2, Mannan-binding lectin; PGA, plasminogen; CRP, C reactive protein; RET4, Retinol-binding protein; F2, prothrombin; TF, transferrin; CBG, transcortin; TTHY, transthyretin; CTRC, α-1-antiquimotripsin; A1AT, α-1-antitripsin; ORM1, α-1-glucoproteín; A2M, y α-2-macroglobulin.

Regarding the APP network, COVID-19 positive patients in cohort 1 present a significant decrease in amyloid related proteins such as serum amyloid components P and A (APCS and SAA, respectively) and retinol-binding protein (RET4). Similar trend occurs in haptoglobin (HP) and α-1-acid glycoprotein (ORM1), proteins related with iron metabolism and blood transport of biomolecules. Related to the coagulation cascade, a deficiency of factor VIII (F8) has been also detected. This is a key factor for the activation of the complete coagulation cascade and also the rest of the interconnected pathways (as depicted in **Figure 3**). In contrast, an increment in serum albumin (ALB) and transferrin (TF) was detected in these patients compared to control subjects.

Considering the different cascades and physiological processes represented in the complexity of acute-phase reactants, a global view of their deregulation in patients with COVID-19 can be depicted (**Figure 3**).

Up- and down variations on APP levels seem reasonable for a pathogen infection and associated tissue damage by ARDS. Specifically, it can be observed how there is a dysregulation in specific proteins of each of the cascades and processes collected among the APPs. The interconnection between proteins and signaling cascades make it evident that as a whole, there is a generalized dysregulation among the APPs. On the other hand, comparing this pattern of proteins obtained in patients with COVID-19, it is striking that we obtain a network of APPs different (even contrary) to that expected in a situation of inflammation and infection where proteins like ALB usually appear depleted in plasma and was found to be enhanced in the studied ARDS patients. By contrast SAA and APCS tend to be elevated in an infectious process and in this screening, they are observed lower than in controls (38).

#### 3.2.1 AAB Profile in COVID-19-Associated ARDS Patients

**Figure 4:**
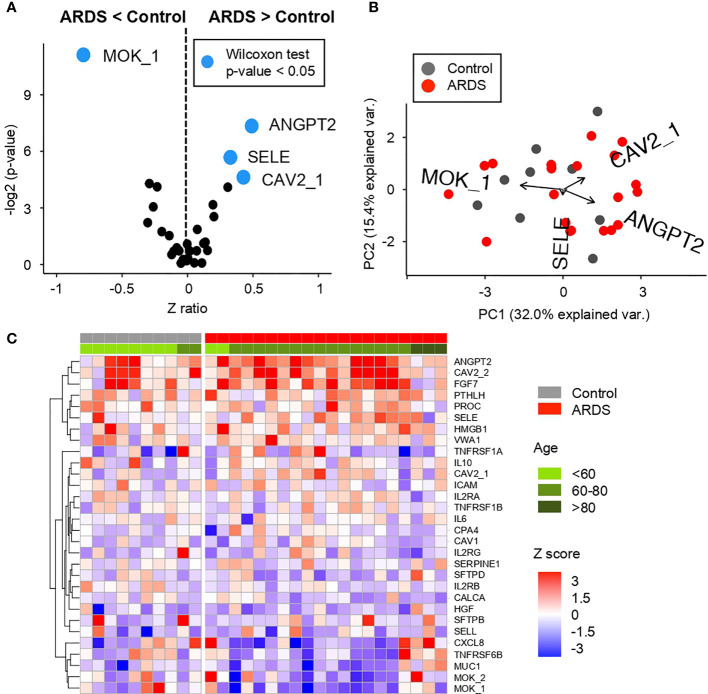
Comparative analysis of the Z scores between control and ARDS patients in the cohort 1 obtained from the microarray for AABs **(A)** Volcano plot summarizing the statistical significance of Z score ratios between control and ARDS patients in the cohort 1 (X-axis). The difference between means was evaluated applying Wilcoxon Rank Sum test (Y-axis). AABs showing statistically significant mean differences are highlighted in blue and larger dots. **(B)** Canonical biplot representing the PCA of the microarray for AABs employed in the cohort 1. Dots represent samples and vectors the AAB variable contribution to the first two principal components at X and Y-axes. The biplot only highlights the most exemplary AAB variables. **(C)** Heat map describing Z score values obtained from the microarray for AABs in cohort 1. Samples are separated by clinical symptoms (gray and red labeled columns) and ordered according to patient age (green color scale).

Considering an exploratory analysis of the AAB profile in cohort 1 (n = 30), differences can be found between ARDS positive (ADSR+) and ARDS negative (control) patients (ADSR−). A total of 30 AABs (**Table S4**) corresponding to cytokines, tissue damage, extracellular matrix proteins, lung tissue proteins, and antigenic proteins associated with other pathologies associated with lung tissue damage, among others, were studied for both groups of patients (**Figure 4**).

Of these AABs, three of them—ANGPT2, SELE, and CAV2—are detected in ARDS+ patients with a highly significant difference (**Figure 4A** and **Figure S4**) (Wilcoxon test p-value <0.05) over ARDS− patients. In contrast, the highly significant difference (Wilcoxon test p-value <0.05) in ADSR− patients is found in MOK detection compared to ARDS+ patients.

These differences in AABs profiles are displayed on **Figures 4A, B**, where it is shown that ANGPT2, SELE, and CAV2 (proteins involved in vascular remodeling, cytokine-activated vascular adhesion, cell growth control and apoptosis, respectively) clustered a group of patients in comparison with MOK (related to inflammation in innate immunity) that is detected only in controls.

Once the most significant differences between patient groups have been identified, a clear correlation is observed between the AABs profiles in ARDS-positive patients according to the age. It is correlated that the higher number of AABs is detected in elderly patients, as it might be expected in previously reported studies about auto-immunity (18, 24). This is observed in **Figure 4C**, where the distribution of proteins is mainly by age group. A high presence of AABs can be observed especially in patients between 60 and 80 years. In this age range, as shown both in **Figures S5A, B**, we found proteins with higher expression such as ANGPT2, CAV2, FGF7, and PROC. In contrast, in patients older than 80 years, a highly significant difference (Wilcoxon test p-value <0.05) is observed in the AABs against of MUC1, TNFRSF6B, CXCL8, and MOK.

In order to further investigate the relationship of both screenings performed for AABs and APPs, a Spearman correlation between both profiles in patients with ARDS has been performed (**Figure S6**). This joint bioinformatics analysis has resulted in positive and negative correlations between some AABs and APPs, strengthening the differential humoral profile of ARDS patients.

### 3.3 Evaluation of AAB Profiling Across COVID-19 Severity

**Figure 5:**
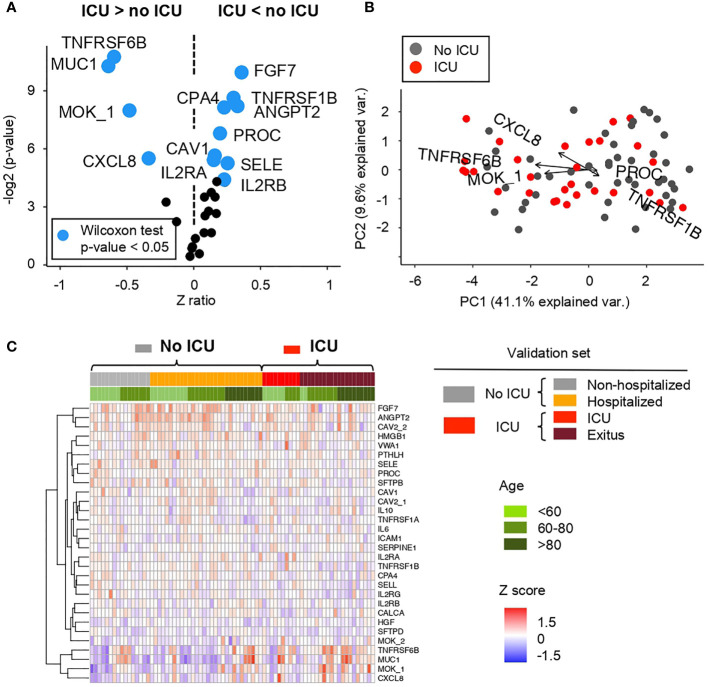
Comparative analysis of the Z scores between patients showing mild and severe ARDS symptoms in the cohort 2 obtained from the microarray for AABs **(A)** Volcano plot summarizing the statistical significance of Z score ratios between patients with mild symptoms or hospitalized (no ICU) and patients admitted to the ICU and/or deceased in the cohort 2 (X-axis). The difference between means was evaluated applying Wilcoxon Rank Sum test (Y-axis). AABs showing statistically significant mean differences are highlighted in blue and larger dots. **(B)** Canonical biplot representing the PCA of the microarray for AABs employed in the cohort 2. Dots represent samples and vectors the AAB variable contribution to the first two principal components at X and Y-axes. The biplot only highlights the most exemplary AAB variables. **(C)** Heat map describing Z score values obtained from the microarray for AABs in the cohort 2. Samples are ordered according to clinical symptoms and patient age (green color scale). Samples grouped under No ICU category include patients with mild symptoms or hospitalized -columns labeled in gray or orange color in the heat map and summarized in gray in the biplot-. UCI group include patients admitted to the ICU and/or deceased -columns labeled in red or brown color in the heat map and summarized in red in the biplot.

Once AABs profile are observed, we explored if this profile is correlated with the severity of the disease. Therefore, it evaluated this AAB profile in a larger cohort of COVID-19 positive patients (n = 76), which are divided in four groups of patients depending on the severity of the pathology (**Figure 5** and **Figure S7**).

Regarding the severity, in order to compare a greater number of AABs expressed in patients with mild and higher severity, two differentiated groups of patients were made. On the one hand, ‘No ICU’, includes non-hospitalized and hospitalized patients. On the other hand, ‘ICU’, includes ICU and exitus individuals.

Between these two groups, significant differences in AAB profiles (Wilcoxon test p-value <0. 05) are observed as shown in the **Figures 5A, B** and **Figure S6A** where AAB against TNFRSF6B (tumor necrosis factor), MUC1 (prognostic lung tumor marker), MOK and CXCL8 (involved in inflammation) are higher in the ‘ICU’ group; while the detection of FGF7 (involved in cell growth and tissue repair), TNFRSF1B (tumor necrosis factor), CPA4 (involved in proteolysis), ANGPT2 (involved in vascular remodeling), PROC (involved in blood clotting), CAV1 (cell cycle regulation), SELE (involved in inflammation), IL2RA and IL2RB (involved in extracellular proteolysis) is higher in the ‘no ICU’ group (**Figures 5A, B**).

**Figure 5C** and **Figure S6B** show the distribution of AABs based on the prognosis of patients, separated into four groups, ranging from low to high severity. Several AABs such as MUC1 or TNFRSF6B, among others, are significantly detected in patients with a more severe pathology. In addition, as previously indicated in cohort 1, a higher expression is observed in elderly patients. On the contrary, FGF7 and ANGPT2, among other AABs, are also significantly detected mostly in patients with lower or minimal severity.

Furthermore, AAB proteins showing largest Z ratios at AAB profiling in **Figure 5A**, were used to define a series of Random Forest (RF) models to classify ARDS COVID-19 patient severity. The performance of RF models at classifying patients admitted to ICU was assessed by calculating the Area Under the Curve (AUC) (**Figures S8, S9**). The combination of TNFRSF6B, MUC1, MOK, and CXCL8 shows an AUC = 0.687, with a 63% of sensitivity and a 76.7% of specificity (**Figure S7A**). However, the combination of AAB proteins significantly decreased at patients admitted to ICU—IL2RB, SFTPD, TNFRSF1B, and AGPT2—returned the RF model with best performance—AUC = 0.8– (**Figure S8**), with 87% of sensitivity and 63.3% of specificity.

## 4 Discussion

In recent months, the study of SARS-COV-2 at all levels (genomic, transcriptomic, proteomic) has been fundamental in understanding the clinical–epidemiological characteristics of COVID-19.

It is estimated that 90% of adults have antibodies against to CoV-NL63, CoV-HKU1, CoV-229E, and CoV-OC43; which are similar virus to SARS-CoV-2 in terms of transmission and replication mechanisms and processes (3). For this reason, in this study, antigenic multiplex assay for CoVs was performed to compare and correlate with the SARS-CoV-2 antigen response. The, it was confirmed that both controls and COVID-19 positive patients present antibodies to several virus antigen proteins and/or all four of the previously circulating CoVs. Furthermore, the presence of antibodies to other coronaviruses does not appear to be related either to the presence of antibodies to SARS-CoV-2, or to being ± SARS-CoV-2 infected. Hence, it can be concluded that previous exposure to other coronaviruses is not affecting the onset of ASDR.

It is known that the vast majority of patients at ICU with severe pneumonia and later ARDS are known to be infected with respiratory viruses. However, the direct pathophysiological link between ARDS and respiratory viruses is still unknown; therefore, there is an urgent need to uncover the mechanistic underpinnings of this process and also the need for the prognostic biomarkers. Patients infected by SARS-CoV, SARS-CoV-2, and MERS-CoV (highly pathogenic viruses), can suffer from severe acute lung injury or ARDS (1, 39). Herein, it is not observed a direct relation between COVID-19 associated ARDS diagnosis and previous infection by CoV-NL63, CoV-HKU1, CoV-229E, and CoV-OC43 as detected by antibodies against these CoVs (40).

With respect to APPs, a novel multiplex assay has been designed and successfully tested to simultaneously screen multiple patients; thus, this screening has allowed us to establish a pattern of ARSD-related acute phase reactants in COVID-19. These APPs can contribute to a better patient diagnosis, prognosis and also helping to complete the clinical picture of the patient for a better stratification and therapeutical options. Many of the APPs (ALB, ORM, CRP among others) appear in the routine clinical analysis (by conventional immunoassays) of hospitalized patients and can be applied to stratify COVID-19 patients according to the severity (41). If the diagnosis of the disease is taken further, these parameters also play an important role in the application of the most appropriate therapeutical strategy. So far, the current treatment indicated for these patients is based on steroidal anti-inflammatory drugs such as dexamethasone (that target the inflammatory response in a non-specific manner) (42). With the study of APPs in a more detailed way, a more targeted and effective anti-inflammatory treatment for patients could be achieved according to particular APPs profiles and its alterations. Thus, APP profile might have a predictive or prognosis value for ARDS-related patients.

Multiple studies have focused on the investigation of COVID-19 AABs and their associated pathologies because they may be triggers for the development of autoimmune and/or inflammatory dysregulation (43). Bearing in mind these results, patients with severe COVID-19 infection have more than just an overactive immune response, their B cells seem to produce AABs. In SARS-COV-2 infection, dysregulation of the immune system can trigger an imbalance of cytokines and immune cell activation. This uncontrolled production and release of proinflammatory cytokines and chemokines can trigger extensive tissue damage as observed in other autoimmune diseases (44). In addition, MHC molecules are essential for antigen presentation and T cell activation. The MHC locus is highly polymorphic, and HLA-B is the most polymorphic locus in the human genome. The MHC molecule determines the epitope presented to the T cell. It has been suggested that some MHC molecules can present viral peptides with epitopes very similar to their own peptides, which may lead to the activation of autoreactive T cells. Variations in the MHC locus are also closely related to many different autoimmune diseases (7, 45, 46) given dysregulation, identification of AABs in COVID-19 and its associated pathologies, as we have observed in our results, have also been demonstrated in other studies about the diversity (47), frequency and suggested function of these AABs (48).

Nowadays, it is known that autoantibodies play a key role in triggering the inflammation responsible for organ damage. ARDS is caused by a wide variety of infectious or inflammatory stimuli in the lung that may originate from direct injury as in pneumonias. The pathological features of ARDS are diffuse alveolar damage manifested by alteration of the capillary interface, and also accumulation of immune cells (innate and adaptive) and protein-rich exudates in the alveolar spaces (14). Likewise, when the inflammation persists in severe cases as it exists in ADSR pathology, it can also generate a sharp drop in APPs, generating an “immunosuppressive” systemic situation that would favor autoantibodies to have more damaging potential. Dysregulation in the humoral response reflected in APPs may result in dysregulation leading to autoimmune-like alterations. Multiple studies have focused on the investigation of autoantibodies described in COVID-19 patients and their associated pathologies (43). Considering these findings, patients with severe COVID-19 infection may have a picture of an overactive immune response in terms of antibody production that would also correlate with the exhausted leukocyte tendency noted above. Dysregulation of the immune system can trigger an imbalance of cytokines and activation of immune cells. This uncontrolled production and release of proinflammatory cytokines and chemokines may trigger extensive tissue damage, as seen in other autoimmune diseases and could be the key to understanding the inflammatory and sepsis processes of this disease (44).

Compared to previous relevant studies regarding the presence of autoantibodies in COVID-19 disease, the patients studied by Bastard et al. presented a percentage of about 10% of presence of autoantibodies against INF type 1 in the case of severe COVID-19 patients (49). With this work, the authors analyzed parameters such as sex or age and were able to correlate it with the severity of the cohorts studied, concluding that adaptive autoimmunity impairs innate and intrinsic antiviral immunity. In this study, in the correlation of the presence of autoantibodies and age, our data are in agreement with the reported work. Moreover, here, we have been able to discriminate severity with an autoantibodies profile presented in all studied cohorts.

In this regard, our results show AABs previously described as a function of pathology severity related to tissue and vascular damage such as MOK1 (50), antigenic proteins associated with lung damage such as MUC1 (51) including pro-inflammatory and inflammatory cytokines such as TNF, and interleukins like CXCL8 involved in the cytokine cascade and related with sepsis and septic shock (37, 52). In addition, our results show a higher amount of AABs in patients with advanced age as demonstrated in studies of other autoimmune pathologies as there is an enhancement of autoimmunity in immunosenescence development (53–56). Overall, these results show an AAB profile that discriminate ± ARDS patients and their severity (**Figures S8, S9**). Furthermore, this determined profile is mostly common in elderly patients, which correlate with the disease outcome and prevalence.

## 5 Conclusions

Monitoring the immune responses in COVID-19 associated ARDS patients help with predicting the disease severity. In fact, many therapeutics target the immune response; mainly the dysregulated hyper-inflammatory state that occurs in some COVID-19 patients. Nevertheless, blood-derived signatures of COVID-19 severity are diverse from lymphopenia, immune suppression, interferon driven immunopathology, T cell activation and exhaustion or immune senescence. Additionally, patients with severe COVID-19 infection have more than just an overactive immune response; their B-cells seem to produce auto-antibodies. In this study, several AABs have been identified in ARDS patients targeting the cytokines, chemokines, glycoproteins, and phospholipoproteins. In addition, it has been possible to identify differential patterns of AABs associated with lung damage depending on the severity of the patients studied. The detection of these AABs could open novel hallmarks to monitor infection dynamic and evolution. In this sense, the degree to which autoimmunity contributes to either mild, acute or severe COVID-19 is still not fully understood and further analysis and characterizations are required to provide novel insights in the disease. Furthermore, it is demonstrated that affinity proteomics combined with systems biology allows the identification of inflammatory mediators, plasmatic protein cascades and auto-antibodies, as an immune fingerprint which could define potential therapeutic targets and biomarkers of the variable responses to SARS-CoV-2 infection.

## Data Availability Statement

The original contributions presented in the study are included in the article/**Supplementary Material**. Further inquiries can be directed to the corresponding authors.

## Ethics Statement

Each individual gave informed consent prior to entering the study and was subsequently approved by the HUS ethics committee.

## Author Contributions

PJ-V performed autoantibodies profile. AL-V performed antigen profile. MG-V, QL, and JR performed bioinformatics analysis. A-PH performed acute-phase protein profile. RH, AF, FC, and JL performed cohort characterization and sample analyses. PJ-V, AL-V, MG-V, A-PH, and MF: writing—original draft. Supervision: MF. Conceptualization: JL, RH, AF, and MF. Funding acquisition: MF. All authors contributed to the article and approved the submitted version.

## Funding

We gratefully acknowledge financial support from the Spanish Health Institute Carlos III (ISCIII) for the grants: FIS PI14/01538, FIS PI17/01930, and CB16/12/00400. This research work was also funded by the European Commission – NextGenerationEU, through CSIC's Global Health Platform (PTI Salud Global) We also acknowledge Fondos FEDER (EU) and Junta Castilla-León (COVID19 grant COV20EDU/00187). The Proteomics Unit belongs to ProteoRed, PRB3-ISCIII, supported by grant PT17/0019/0023, of the PE I + D + I 2017-2020, funded by ISCIII and FEDER. AL-V is supported by the VIII Centenario-USAL PhD Program. PJ-V is supported by the JCYL PhD Program “Nos Impulsa-JCYL” and scholarship JCYL-EDU/601/2020. EXOHEP-CM S2017/BMD3727 by Comunidad de Madrid and Fondos FEDER (to JL and RH) and PI19/01091 by ISCIII (to RH).

## Conflict of Interest

The authors declare that the research was conducted in the absence of any commercial or financial relationships that could be construed as a potential conflict of interest.

## Publisher’s Note

All claims expressed in this article are solely those of the authors and do not necessarily represent those of their affiliated organizations, or those of the publisher, the editors and the reviewers. Any product that may be evaluated in this article, or claim that may be made by its manufacturer, is not guaranteed or endorsed by the publisher.
