# Supplementary material for: SARS-CoV-2 Infection Triggers Auto-Immune Response in ARDS
Source: Front Immunol. 2022 Jan 28;13:732197. doi: 10.3389/fimmu.2022.732197 (PMC8831226; doi:10.3389/fimmu.2022.732197)
Supplement: Supplementary file 1 [file DataSheet_1.docx]

***Supplementary Material***

# Supplementary tables

Table S1. Clinical resume characteristics of subjects from cohort 1.

| **Characteristics** | | **ARDS Positive (n=20)** | **ARDS Negative (n=10)** | **Total Cases (n=30)** |
| --- | --- | --- | --- | --- |
| **Age (Years)** | ***Minimum*** | 55 | 18 | 18 |
|  | ***Maximum*** | 92 | 65 | 92 |
|  | ***Median*** | 70 | 39 | 59 |
| **Gender** | ***Female*** | 10 (50 %) | 3 (30 %) | 13 (43.3%) |
|  | ***Male*** | 10 (50 %) | 7 (70 %) | 17 (56.7%) |
| **SARS-Cov2 Serological test** | | | | |
| **Positive** | ***IgG*** | 16 (80 %) | - | 16 (53.3 %) |
|  | ***IgM*** | 10 (50 %) | - | 10 (33.3 %) |
|  | ***Both Ig*** | 6 (30 %) | - | 6 (20%) |
|  | ***Only IgG*** | 10 (50 %) | - | 10 (33%) |
|  | ***Only IgM*** | 4 (20 %) | - | 4 (13.3 %) |
| **SARS-Cov2 PCR test** | | 19 (95 %) | - | 19 (63.3 %) |

*IgG and IgM were studied for the SARS-CoV-2 N protein*

Table S2. Full clinical characteristics of subjects from cohort 1.

| **Subject** | **Age** | **Sex** | **Body Mass Index (BMI)** | **COVID diagnostic** | **COVID PCR test** | **COVID serological test** | **CG-COV-IgG Test Line** | **CG-COV-IgM Test Line** | **Combined** | **Days sample collection after PCR** |
| --- | --- | --- | --- | --- | --- | --- | --- | --- | --- | --- |
| 1 | 67 | F | n.a. | positive | positive | positive | - | + | + | 32 |
| 2 | 55 | M | n.a. | positive | positive | positive | + | + | + | 32 |
| 3 | 69 | M | n.a. | positive | positive | positive | + | - | + | 33 |
| 4 | 69 | F | n.a. | positive | positive | positive | + | - | + | 33 |
| 5 | 64 | F | n.a. | positive | positive | positive | + | - | + | 32 |
| 6 | 89 | M | n.a. | positive | positive | positive | + | + | + | 33 |
| 7 | 92 | F | n.a. | positive | positive | positive | + | - | + | 32 |
| 8 | 71 | M | n.a. | positive | positive | positive | + | ++ | + | 32 |
| 9 | 66 | M | n.a. | positive | positive | positive | + | + | + | 33 |
| 10 | 80 | F | n.a. | positive | positive | positive | - | ++ | + | 33 |
| 11 | 76 | F | n.a. | positive | positive | positive | - | + | + | -2 |
| 12 | 58 | M | n.a. | negative | negative | positive | - | + | + | -2 |
| 13 | 71 | M | n.a. | positive | positive | positive | + | - | + | 30 |
| 14 | 63 | F | n.a. | positive | positive | positive | + | - | + | 30 |
| 15 | 74 | F | n.a. | positive | positive | positive | + | - | + | 30 |
| 16 | 63 | F | n.a. | positive | positive | positive | + | - | + | 30 |
| 17 | 61 | M | n.a. | positive | positive | positive | ++ | - | + | 30 |
| 18 | 67 | M | n.a. | positive | positive | positive | ++ | - | + | 30 |
| 19 | 74 | F | n.a. | positive | positive | positive | ++ | + | + | 30 |
| 20 | 63 | M | n.a. | positive | positive | positive | ++ | + | + | 30 |
| 21 | 46 | M | 28,7 | negative | negative | negative | n.a. | n.a. | n.a. | n.a. |
| 22 | 22 | F | 21,7 | negative | negative | negative | n.a. | n.a. | n.a. | n.a. |
| 23 | 18 | M | 26,2 | negative | negative | negative | n.a. | n.a. | n.a. | n.a. |
| 24 | 47 | M | 35,2 | negative | negative | negative | n.a. | n.a. | n.a. | n.a. |
| 25 | 25 | F | 22,3 | negative | negative | negative | n.a. | n.a. | n.a. | n.a. |
| 26 | 65 | M | 36 | negative | negative | negative | n.a. | n.a. | n.a. | n.a. |
| 27 | 28 | M | 20,6 | negative | negative | negative | n.a. | n.a. | n.a. | n.a. |
| 28 | 63 | M | 19,2 | negative | negative | negative | n.a. | n.a. | n.a. | n.a. |
| 29 | 38,7 | F | 31,8 | negative | negative | negative | n.a. | n.a. | n.a. | n.a. |
| 30 | 36,5 | M | n.a. | negative | negative | negative | n.a. | n.a. | n.a. | n.a. |

M: male; F: female; n.a.: not available; GC: Colloidal Gold Method

Table S3. Clinical resume characteristics of subjects from cohort 2

| **Characteristics** | **ARDS Positive**  **(n=30)** | **ARDS Negative**  **(n=46)** | **Total cases**  **(n=76)** |  |
| --- | --- | --- | --- | --- |
| **Age (years)** | | | | |
| Min | 42 | 31 | 31 |  |
| Max | 89 | 86 | 89 |  |
| Mean | 70 | 60 | 65 |  |
| **Gender** | | | | |
| Female | 12 (40%) | 23 (50%) | 35(46%) |  |
| Male | 18 (60%) | 23 (50%) | 41(54%) |  |
| **COVID-19 severity** | | | |  |
| Non hospitalized | - | 16 (34.8%) | 16 (21%) |  |
| Hospitalized | - | 30 (65.2%) | 30 (39.5%) |  |
| ICU | 10 (33.3%) | - | 10 (13.2%) |  |
| Exitus | 20 (66.6%) | - | 20 (26.3%) |  |

Table S4. Full clinical characteristics of subjects from cohort 2.

| **Subject** | **Age** | **Sex** | **COVID diagnostic** | **COVID severity** | **Symptom onset** | **Comorbidities** |
| --- | --- | --- | --- | --- | --- | --- |
| 1 | 44 | M | yes | Exitus | 9 | yes |
| 2 | 56 | M | yes | Exitus | 9 | yes |
| 3 | 63 | M | yes | Exitus | 6 | yes |
| 4 | 69 | F | yes | Exitus | 7 | yes |
| 5 | 69 | F | yes | Exitus | 10 | yes |
| 6 | 70 | M | yes | Exitus | 10 | yes |
| 7 | 71 | M | yes | Exitus | 8 | yes |
| 8 | 75 | F | yes | Exitus | 8 | yes |
| 9 | 75 | M | yes | Exitus | 6 | yes |
| 10 | 76 | M | yes | Exitus | 7 | yes |
| 11 | 82 | M | yes | Exitus | 11 | yes |
| 12 | 83 | F | yes | Exitus | 10 | yes |
| 13 | 83 | F | yes | Exitus | 7 | no |
| 14 | 83 | F | yes | Exitus | 5 | yes |
| 15 | 84 | F | yes | Exitus | 11 | yes |
| 16 | 86 | F | yes | Exitus | 7 | yes |
| 17 | 87 | M | yes | Exitus | 11 | yes |
| 18 | 88 | F | yes | Exitus | 6 | yes |
| 19 | 89 | F | yes | Exitus | 5 | yes |
| 20 | 89 | M | yes | Exitus | 12 | yes |
| 21 | 42 | M | yes | ICU | 9 | yes |
| 22 | 44 | M | yes | ICU | 8 | yes |
| 23 | 48 | M | yes | ICU | 6 | yes |
| 24 | 52 | M | yes | ICU | 11 | yes |
| 25 | 54 | M | yes | ICU | 9 | yes |
| 26 | 57 | F | yes | ICU | 12 | yes |
| 27 | 62 | M | yes | ICU | 8 | yes |
| 28 | 66 | F | yes | ICU | 8 | yes |
| 29 | 67 | M | yes | ICU | 7 | yes |
| 30 | 71 | M | yes | ICU | 8 | yes |
| 31 | 31 | F | yes | Hospitalised | 7 | yes |
| 32 | 32 | F | yes | Hospitalised | 9 | yes |
| 33 | 35 | M | yes | Hospitalised | 8 | no |
| 34 | 36 | M | yes | Hospitalised | 7 | yes |
| 35 | 39 | M | yes | Hospitalised | 11 | yes |
| 36 | 39 | F | yes | Hospitalised | 10 | yes |
| 37 | 40 | M | yes | Hospitalised | 11 | yes |
| 38 | 42 | F | yes | Hospitalised | 8 | yes |
| 39 | 43 | M | yes | Hospitalised | 8 | yes |
| 40 | 43 | F | yes | Hospitalised | 7 | yes |
| 41 | 61 | F | yes | Hospitalised | 9 | yes |
| 42 | 62 | F | yes | Hospitalised | 12 | yes |
| 43 | 62 | M | yes | Hospitalised | 9 | yes |
| 44 | 63 | M | yes | Hospitalised | 10 | yes |
| 45 | 63 | M | yes | Hospitalised | 9 | yes |
| 46 | 64 | M | yes | Hospitalised | 5 | yes |
| 47 | 64 | M | yes | Hospitalised | 8 | yes |
| 48 | 64 | M | yes | Hospitalised | 9 | yes |
| 49 | 66 | M | yes | Hospitalised | 7 | yes |
| 50 | 66 | F | yes | Hospitalised | 10 | yes |
| 51 | 81 | F | yes | Hospitalised | 5 | yes |
| 52 | 81 | F | yes | Hospitalised | 5 | yes |
| 53 | 83 | F | yes | Hospitalised | 9 | yes |
| 54 | 83 | M | yes | Hospitalised | 7 | yes |
| 55 | 83 | M | yes | Hospitalised | 7 | yes |
| 56 | 85 | F | yes | Hospitalised | 8 | yes |
| 57 | 85 | M | yes | Hospitalised | 7 | yes |
| 58 | 85 | F | yes | Hospitalised | 9 | yes |
| 59 | 85 | M | yes | Hospitalised | 9 | yes |
| 60 | 86 | F | yes | Hospitalised | 8 | yes |
| 61 | 37 | M | yes | Not Hospitalised | 7 | yes |
| 62 | 41 | F | yes | Not Hospitalised | 8 | yes |
| 63 | 42 | F | yes | Not Hospitalised | 5 | yes |
| 64 | 46 | M | yes | Not Hospitalised | 7 | yes |
| 65 | 46 | F | yes | Not Hospitalised | 10 | yes |
| 66 | 49 | M | yes | Not Hospitalised | 6 | yes |
| 67 | 53 | F | yes | Not Hospitalised | 5 | yes |
| 68 | 58 | F | yes | Not Hospitalised | 10 | yes |
| 69 | 63 | F | yes | Not Hospitalised | 12 | yes |
| 70 | 63 | M | yes | Not Hospitalised | 6 | yes |
| 71 | 64 | F | yes | Not Hospitalised | 7 | yes |
| 72 | 68 | F | yes | Not Hospitalised | 8 | yes |
| 73 | 68 | M | yes | Not Hospitalised | 6 | yes |
| 74 | 70 | M | yes | Not Hospitalised | 8 | yes |
| 75 | 76 | M | yes | Not Hospitalised | 11 | yes |
| 76 | 75 | F | yes | Not Hospitalised | 10 | yes |

M: male; F: female; ICU: Intensive Care Unit

Table S5: Antibodies used in APPs array and ADRS array.

| **Antibody** | **Reference** | **Supplier** |
| --- | --- | --- |
| Anti-Chymotrypsin Antibody* | ab35694 | Abcam (Cambridge, UK) |
| Anti-Serum Amyloid P/SAP Antibody* | ab45151 |  |
| Recombinant Anti-C3 Antibody* | ab200999 |  |
| Anti-Mannan Binding Lectin/MBL antibody* | ab189856 |  |
| Recombinant Anti-Ferritin Antibody* | ab75973 |  |
| Anti-Serum Amyloid-A Antibody* | ab190802 |  |
| Anti-Haptoglobin Antibody* | ab13429 |  |
| Transthyretin Polyclonal Antibody* | 856302 | Biolegend (San Diego/CA, USA) |
| Anti-Retinol Binding Protein RBP Antibody* | MCA4655 | BioRad (Hercules/CA, USA) |
| Complement C4 Polyclonal Antibody* | AHP1753 |  |
| Ceruloplasmin Antibody* | 1940-0004 |  |
| Anti-Transferrin Antibody* | VMA00578 |  |
| Alpha-2-Macroglobulin Antibody* | 5850-0004 |  |
| Anti-GST Antibody | 27457701V | GE Healthcare (Little Chalfont, Buckinghamshire, UK) |
| Peroxidase-conjugated Goat Anti-Human IgG | 109-035-098 | Jackson Immunoresearch (Cambridge, UK) |
| Peroxidase-conjugated Sheep Anti-Mouse IgG | 515-035-062 |  |
| Anti-Biotin Antibody | 200-02-211 |  |
| Mouse Serpin A6 Antibody (Transcortin)* | MAB4065 | RD Systems (Minneapolis/MN, USA) |
| Anti-Alpha 1 Acid Glycoprotein/AGP Antibody* | MAB3694 |  |
| Anti-Albumin Antibody* | A0433 | Sigma-Aldrich (St. Louis/MO, USA) |
| Anti-Von Willebrand Factor Antibody* | F3520 |  |
| Anti-C Reactive Protein Antibody* | 11250-R106 | Sino Biological (Wayne/PA, USA) |
| Anti-Plasminogen Antibody* | PA5-14196 | Thermo Scientific (Rockford/IL, USA) |
| Anti-Thrombin Antibody* | PA1-43040 |  |
| Factor VIII Polyclonal Antibody* | PA1-43045 |  |
| Fibrinogen Polyclonal Antibody* | PA1-85429 |  |
| Prothrombin Polyclonal Antibody* | PA5-77976 |  |
| Anti-alpha 1 Antitrypsin Antibody* | 711079 |  |

*Antibodies used for detecting APPs

Table S6: Content of NAPPA-ARDS Arrays

| Official Symbol provided by HGNC | Official Full Name provided by HGNC | Gene ID NCBI | Uniprot ID |
| --- | --- | --- | --- |
| ANGPT2 | angiopoietin 2 | 285 | O15123 |
| CALCA | calcitonin related polypeptide alpha | 796 | P01258 |
| CAV1 | caveolin 1 | 857 | Q03135 |
| CAV2_1 | caveolin 2 | 858 | P51636 |
| CAV2_2 | caveolin 2 | 858 | P51636 |
| CPA4 | carboxypeptidase A4 | 51200 | Q9UI42 |
| FGF7 | fibroblast growth factor 7 | 2252 | P21781 |
| HGF | hepatocyte growth factor | 3082 | P08581 |
| HMGB1 | high mobility group box 1 | 3146 | P09429 |
| ICAM1 | intercellular adhesion molecule 1 | 3383 | P05362 |
| IL10 | interleukin 10 | 3586 | P22301 |
| IL2RA | interleukin 2 receptor subunit alpha | 3559 | P01589 |
| IL2RB | interleukin 2 receptor subunit beta | 3560 | P14784 |
| IL2RG | interleukin 2 receptor subunit gamma | 3561 | P31785 |
| IL6 | interleukin 6 | 3569 | P05231 |
| CXCL8 | C-X-C motif chemokine ligand 8 | 3576 | P10145 |
| MUC1 | mucin 1, cell surface associated | 4582 | P15941 |
| SERPINE1 | serpin family E member 1 | 5054 | P05121 |
| PROC | protein C, inactivator of coagulation factors Va and VIIIa | 5624 | P04070 |
| PTHLH | parathyroid hormone like hormone | 5744 | P12272 |
| MOK_1 | MOK protein kinase | 5891 | Q9UQ07 |
| MOK_2 | MOK protein kinase | 5891 | Q9UQ07 |
| SELE | selectin E | 6401 | P16581 |
| SELL | selectin L | 6402 | P14151 |
| SFTPB | surfactant protein B | 6439 | P07988 |
| SFTPD | surfactant protein D | 6441 | P35247 |
| TNFRSF1A | TNF receptor superfamily member 1A | 7132 | P19438 |
| TNFRSF1B | TNF receptor superfamily member 1B | 7133 | P20333 |
| TNFRSF6B | TNF receptor superfamily member 6B | 8771 | O95407 |
| VWA1 | von Willebrand factor A domain containing 1 | 64856 | Q6PCB0 |

HGNC: HUGO Gene Nomenclature Committee

NCBI: National Center for Biotechnology Information

Table S7: Types of Coronaviruses analyzed in antigen profile assay and their characteristics

| Type of Coronavirus | Antibody detected | Protein target |
| --- | --- | --- |
| CoV-229E | Ig Total | S1 |
| CoV-HKU1 | Ig Total | S1 |
| CoV-OC43 | Ig Total | S1 |
| CoV-NL63 | Ig Total | S1 |
| SARS S1 | Ig Total | S1 |
| SARS-CoV2 | Ig Total | RBD |
| SARS-CoV2 | Ig Total | Nucleocapsid |
| SARS-CoV2 | Ig Total | Spike (Trimer) |
| SARS-CoV2 | Ig Total | S1 Protein |

# Supplementary figures


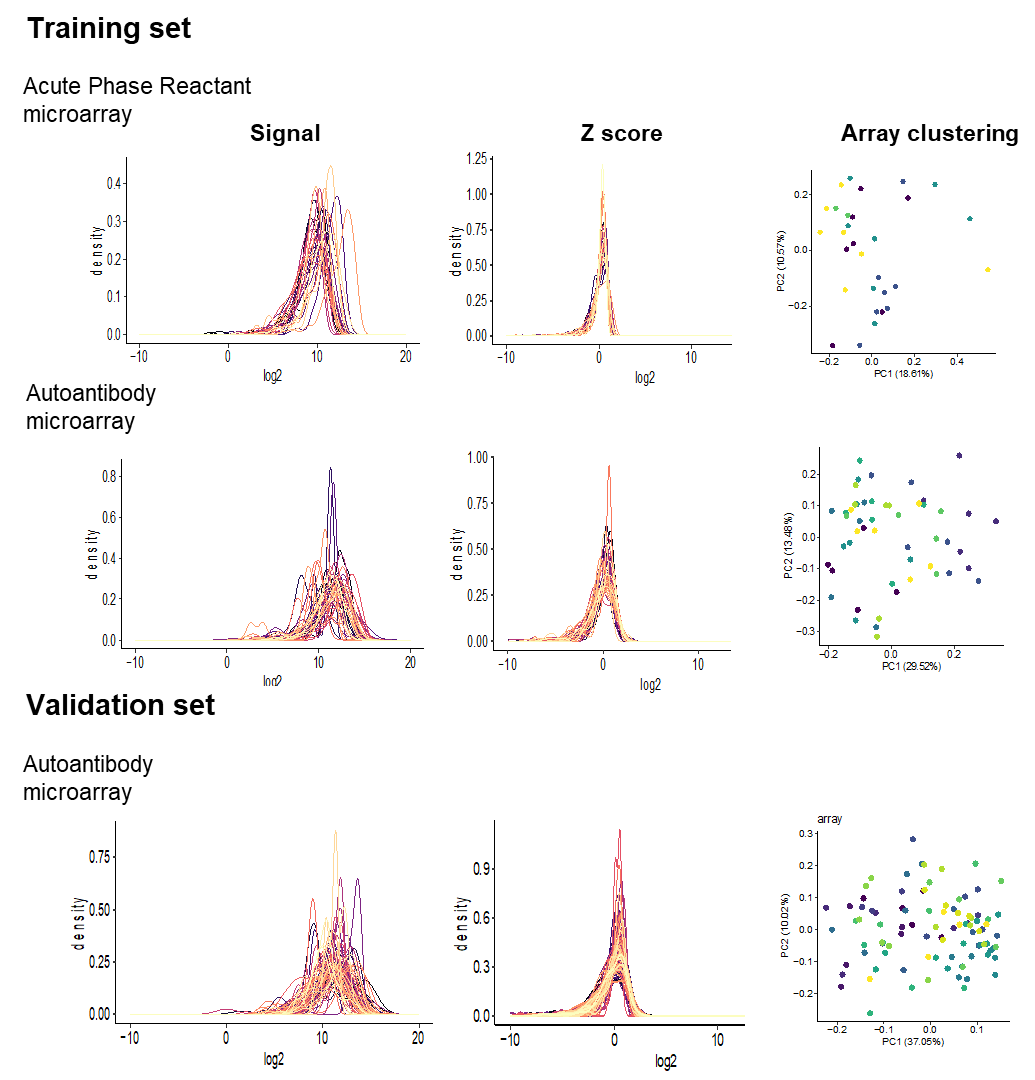


Figure S1: Density plots describing raw and Z score-normalized signal density distribution of each protein microarray (left and center columns). Each density line corresponds to an independent sample. (Right column) Dot plots representing Principal Component Analysis of each protein microarray. Dots represent samples while dot color indicates the localization at the protein microarray (subarray). First row summarizes the outputs retrieved from Acute Phase Reactant microarray (ARFA) employed in the ARDS cohort 1. Second and third rows summarize AAB profile microarray (ARDS) outputs for the ARDS cohort 1 and 2 respectively.


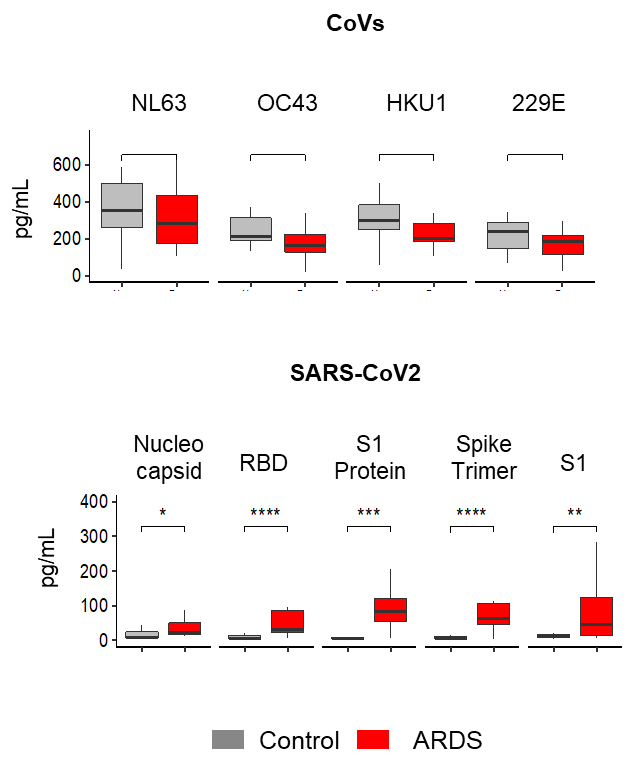


Figure S2: Comparative analysis of the CoV and SARS-CoV-2 antigen concentration at multiplex assay between control and ARDS patients in the cohort 1. Box plots comparing SARS-CoV-2 and other coronaviruses (Cov) antigen concentration (pg/mL) between control (grey) and ARDS patients (red) in the cohort 1. The antigen concentration was quantified through the antigen multiplex assay. The difference between means was evaluated applying Wilcoxon Rank Sum test -p-value symbols; * <0.05, ** < 0.01, *** <0.001 and **** < 0.0001-.


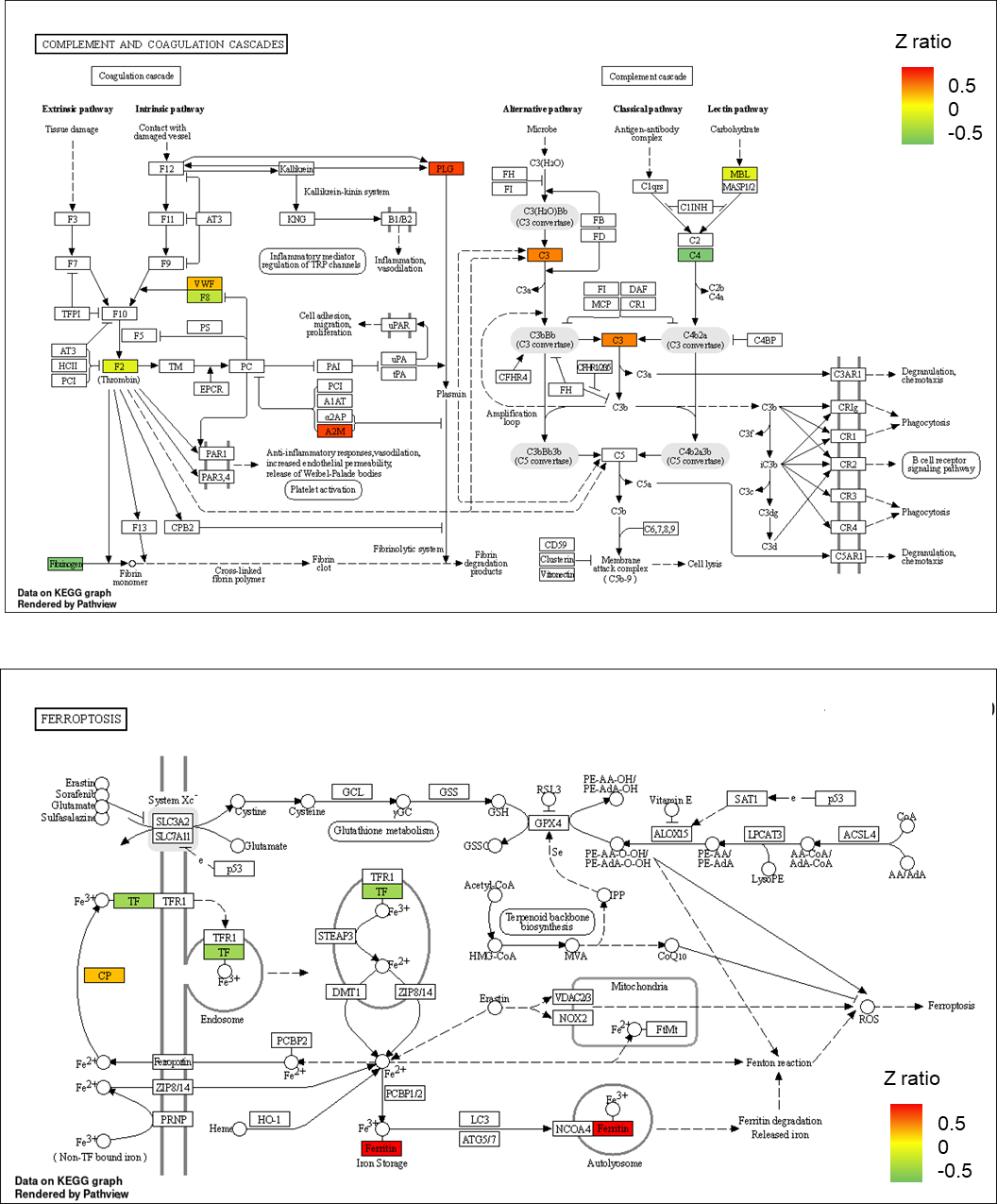


Figure S3: Acute Phase reactant mapping in Complement and coagulation cascades and Ferroptosis KEGG pathways KEGG schematic illustrations of Complement and coagulation cascades and Ferroptosis pathways (hsa04216 and hsa04610). Acute Phase reactants evaluated at the microarray were mapped into KEGG pathways and colored according to Z ratios obtained when comparing the Z score of control and ARDS patients in the cohort 1.


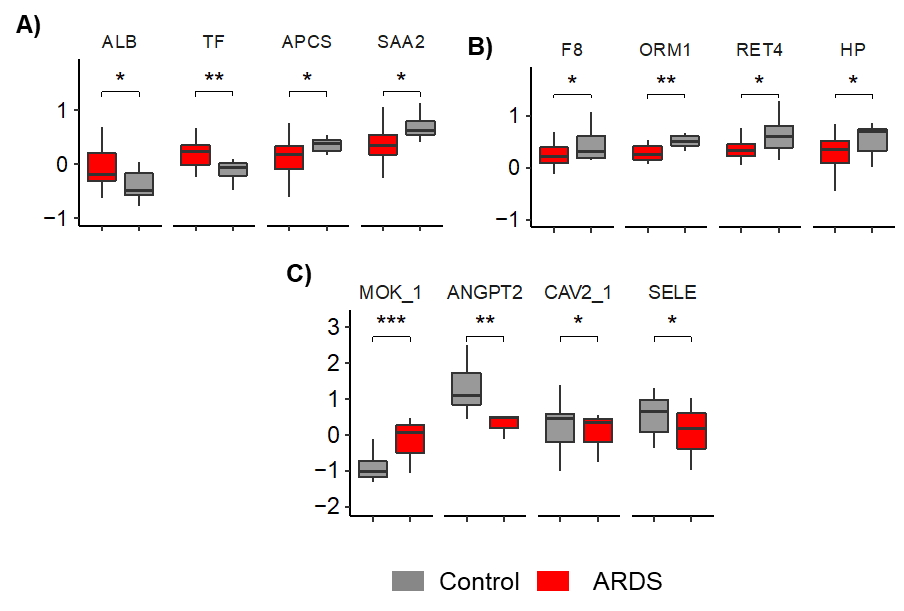


Figure S4: Comparative analysis of the Z scores between control and ARDS patients in the cohort1 obtained from the microarray for Acute Phase reactants Box plots summarizing the significant mean Z score differences between control (grey) and ARDS patients (red) in the microarray for the Acute Phase reactant in 1:500 dilution (A) and 1:5000 (B), and AABs (C). The difference between means was evaluated applying Wilcoxon Rank Sum test -p-value symbols; * <0.05, ** < 0.01, *** <0.001 and **** < 0.0001-.


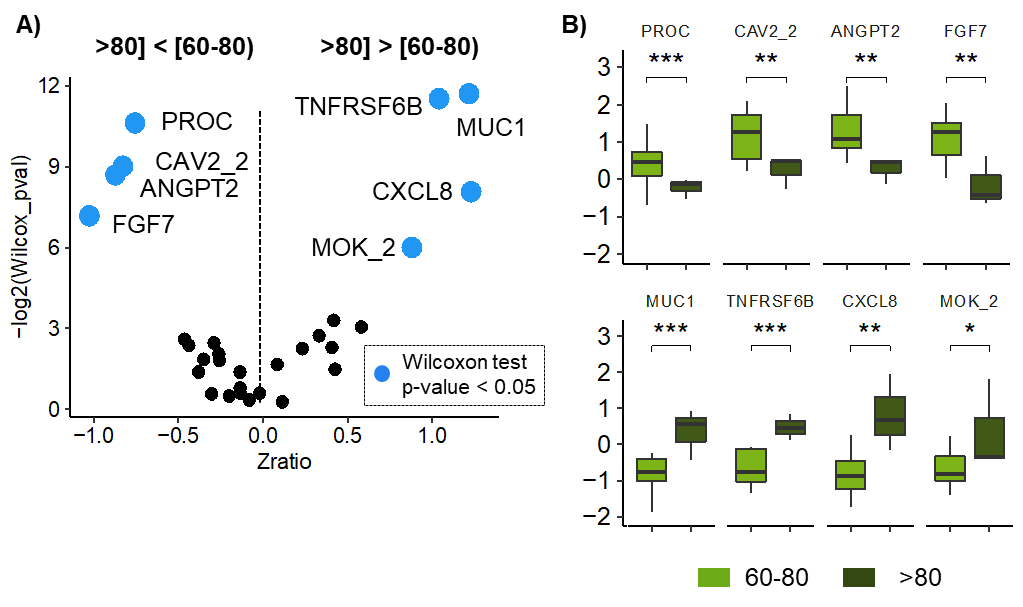


Figure S5: Comparative analysis of the Z scores between age intervals of ARDS patients in the cohort 1 obtained from the microarray for AABs (A) Volcano plot illustrating the Z score ratios of ARDS positive cases when comparing patients between 60-80 years and patients older than 80 years. Proteins showing statistically significant mean differences are highlighted in blue and larger dots. (B) Box plot summarizing the significant mean Z score differences obtained in the Volcano plot. The difference between means was evaluated applying Wilcoxon Rank Sum test -p-value symbols; * <0.05, ** < 0.01, *** <0.001 and **** < 0.0001-.


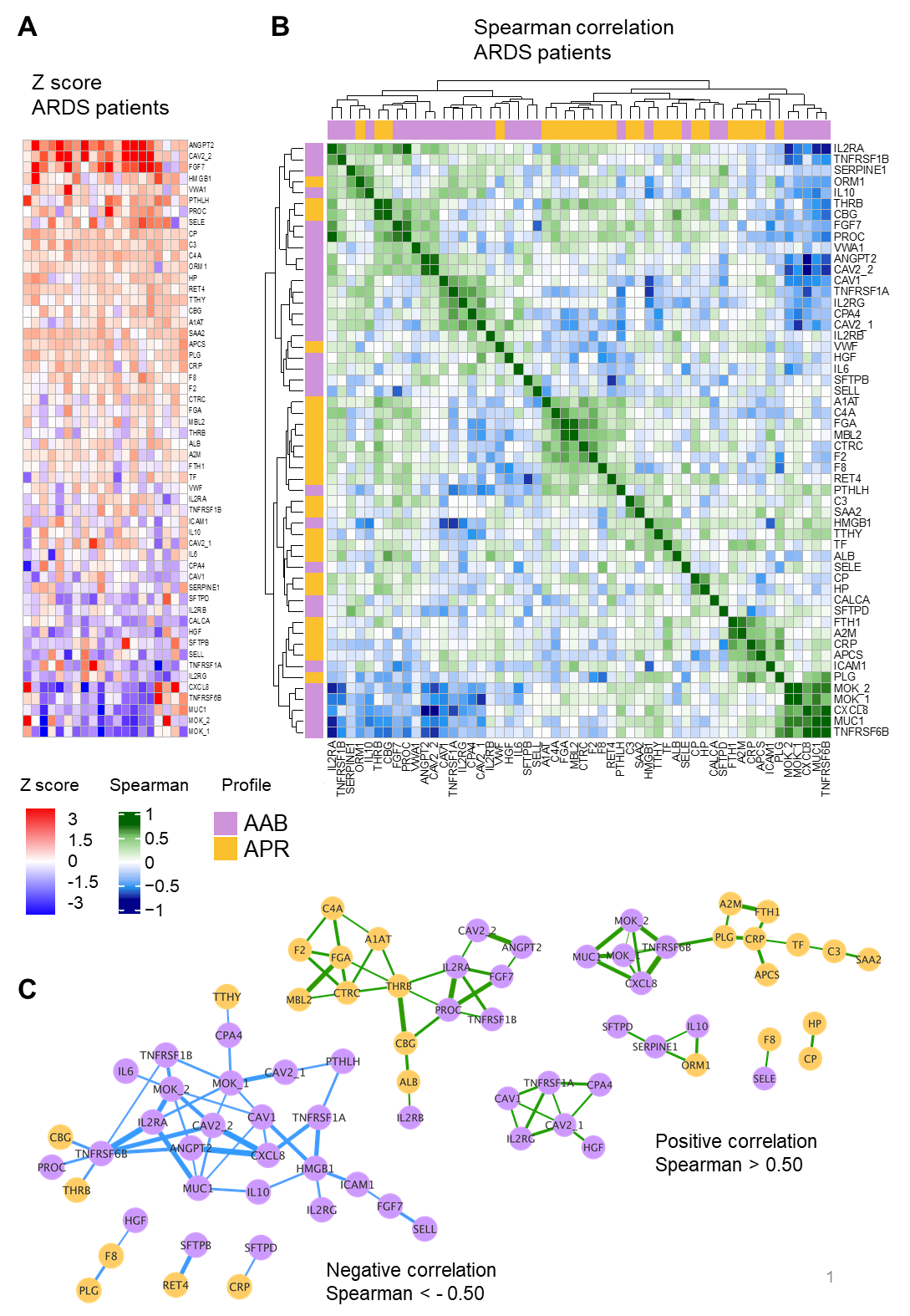


Figure S6: (A) Heat map representing the Z scores obtained in AAB and APR profiles of patients with ARDS. (B) Heat map representing the Spearman correlation between AAB and APR profiles in patients with ARDS. (C) The proteins correlated above a Spearman coefficient higher than absolute 0.5 are then represented in bottom network graphs. AAB and APR proteins are represented in purple and yellow-colored nodes, respectively. Of note, the width and color in network links represent the Spearman coefficient and do not necessarily indicate physical interaction or functional collaboration on particular pathways.


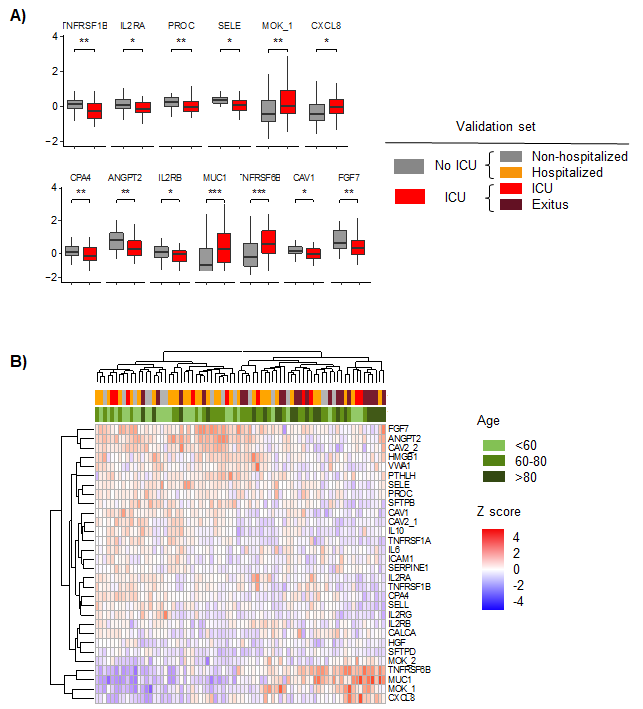


Figure S7. (A) Box plot summarizing the significant mean Z score differences between patients non-admitted to the ICU and patients admitted to the ICU in the cohort 2. Samples grouped under No ICU category include patients with mild symptoms or hospitalized -columns labeled in grey or orange color in the heat map and summarized in grey in the boxplot-. UCI group include patients admitted to the ICU and/or deceased -columns labeled in red or brown color in the heat map and summarized in red in the boxplot-. The difference between means was evaluated applying Wilcoxon Rank Sum test -p-value symbols; * <0.05, ** < 0.01, *** <0.001 and **** < 0.0001-. (B) Heat map describing Z score values obtained from the microarray for AABs in the cohort 2. Sample (row) and AAB (column) unsupervised clustering was performed using Euclidean distance and Complete-linkage clustering method. Green color scale indicates patient age intervals.

**
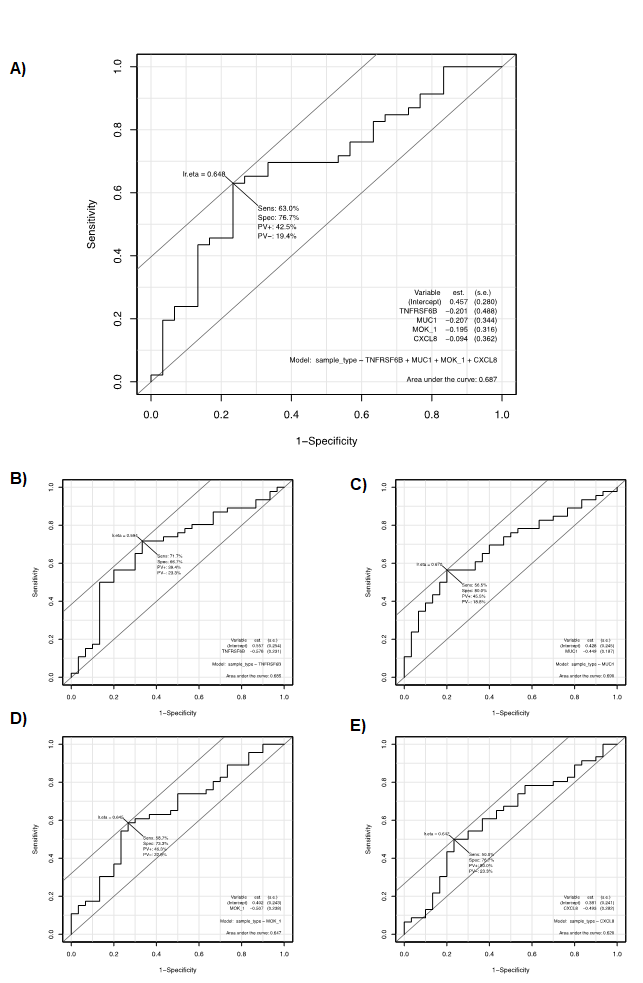
**

Figure S8. Predictive value of AAB profile analysis at COVID-19 ARDS prognosis. (A) Protein microarray profile of AAB for TNFRSF6B, MUC1, MOK_1 and CXCL8 was employed to generate a Random Forest (RF) classification model of ARDS prognosis. RF performance was evaluated by calculating AUC and ROC curves when classifying individuals at cohort 2 with mild symptoms (no ICU) and patients admitted to ICU. (B-E) ROC curves retrieved for RF models defined using only one of the AAB abovementioned. RF and ROC curves were generated using randomForest and EPI R packages -ntry =2,ntree=500-.


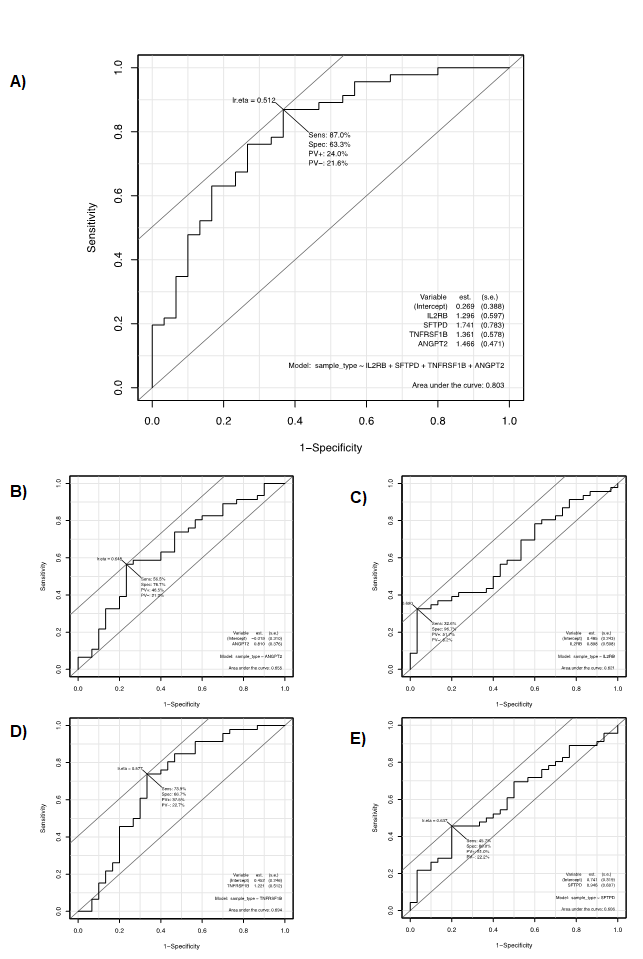


Figure S9. Predictive value of AAB profile analysis at COVID-19 ARDS prognosis. (A) Protein microarray profile of AAB for IL2RB, SFTPD, TNFRSF1B and ANGPT2 was employed to generate a Random Forest (RF) classification model of ARDS prognosis. RF performance was evaluated by calculating AUC and ROC curves when classifying individuals at cohort 2 with mild symptoms (no ICU) and patients admitted to ICU. (B-E) ROC curves retrieved for RF models defined using only one of the AAB abovementioned. RF and ROC curves were generated using randomForest and EPI R packages -ntry=2, ntree=500-.
